# Involvement of endothelins in neuroprotection of valosin-containing protein modulators against retinal ganglion cell damage

**DOI:** 10.1038/s41598-022-20497-w

**Published:** 2022-09-28

**Authors:** Mami Kusaka, Tomoko Hasegawa, Hanako Ohashi Ikeda, Yumi Inoue, Sachiko Iwai, Kei Iida, Akitaka Tsujikawa

**Affiliations:** 1 Department of Ophthalmology and Visual Sciences, Kyoto University Graduate School of Medicine, 54 Kawahara-cho, Shogoin, Sakyo-ku, Kyoto, 606-8507 Japan; 2 Research Fellow of Japan Society for the Promotion of Science, Tokyo, Japan; 3 Medical Research Support Center, Kyoto University Graduate School of Medicine, Kyoto, Japan

**Keywords:** Drug development, Glaucoma

## Abstract

We have previously shown that Kyoto University Substances (KUSs), valosin-containing protein (VCP) modulators, suppress cell death in retinal ganglion cells of glaucoma mouse models through alterations of various genes expressions. In this study, among the genes whose expression in retinal ganglion cells was altered by KUS treatment in the N-methyl-d-aspartic acid (NMDA) injury model, we focused on two genes, endothelin-1 (*Edn1*) and endothelin receptor type B (*Ednrb*), whose expression was up-regulated by NMDA and down-regulated by KUS treatment. First, we confirmed that the expression of Edn1 and Ednrb was upregulated by NMDA and suppressed by KUS administration in mice retinae. Next, to clarify the influence of KUSs on cell viability in relation to the endothelin signaling, cell viability was examined with or without antagonists or agonists of endothelin and with or without KUS in 661W retinal cells under stress conditions. KUS showed a significant protective effect under glucose-free conditions and tunicamycin-induced stress. This protective effect was partially attenuated in the presence of an endothelin antagonist or agonist under glucose-free conditions. These results suggest that KUSs protect cells partially by suppressing the upregulated endothelin signaling under stress conditions.

## Introduction

In the aging society, visual impairment due to glaucoma and age-related macular degeneration is expected to continually increase worldwide^1^. Moreover, there are cases where visual impairment progresses even with the currently established treatments, such as intraocular pressure reduction in glaucoma^2^ or vitreous injection of anti-VEGF drugs in age-related macular degeneration^3^. The possible treatment of ocular diseases, including glaucoma and macular degeneration, might protect retinal nerve cells.

**Figure 1:**
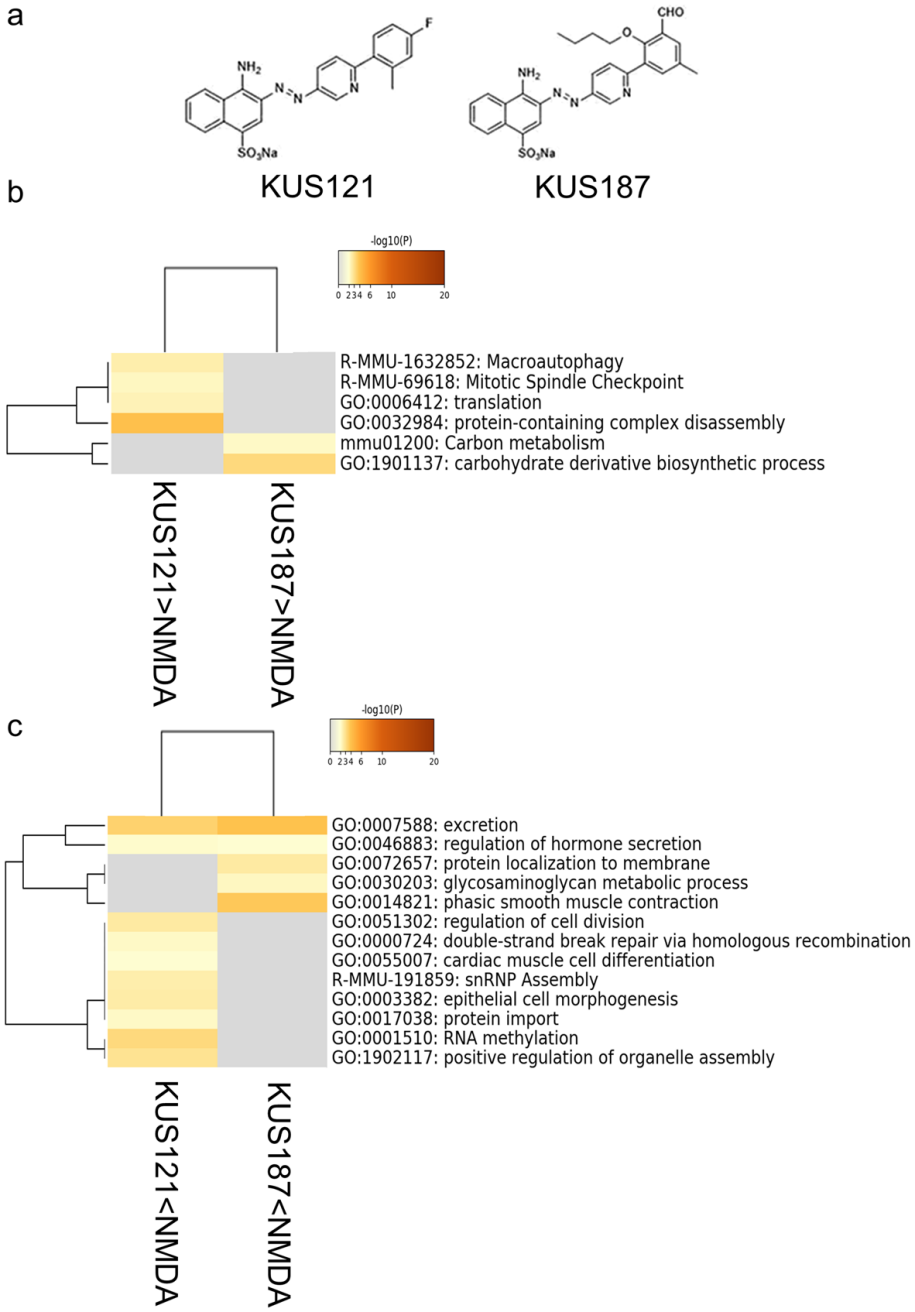
Gene ontology (GO)/pathway analysis of gene clusters. (**a**) Chemical structures of KUS121 and KUS187. (**b**) GO terms and pathways that were significantly related to genes downregulated by NMDA injection and upregulated by KUS treatment. There were no GO terms or pathways which were common between KUS121 and KUS187. (**c**) GO terms and pathways that were significantly related to genes upregulated by NMDA injection and downregulated by KUS treatment. GO terms that were common between KUS121 and KUS187 included excretion (GO: 0007588). KUS121 < NMDA: analysis of genes that were upregulated in NMDA-injected mice retina and downregulated by KUS121 treatment, KUS187  <  NMDA: analysis of genes that were upregulated in NMDA-injected mice retina and downregulated by KUS187 treatment, KUS121 > NMDA: analysis of genes that were downregulated in NMDA-injected mice retina and upregulated by KUS121 treatment, KUS187 > NMDA: analysis of genes that were downregulated in NMDA-injected mice retina and upregulated by KUS187 treatment.

We have previously shown that the novel synthesized Kyoto University Substances (KUSs), which modulate ATPase activity of the valosin-containing protein (VCP), the most abundant soluble ATPase in the cell, protect cells under stress conditions^4^. Several types of KUSs have a common naphthalene-derived structure and exhibit similar physiological activities (Fig. 1a). KUSs have been shown to protect neuronal cells in animal models of glaucoma^5^, retinitis pigmentosa^4,6^, macular degeneration^7^ and retinal artery occlusion^8^. Moreover, a first-in-human clinical trial (phase 1/2) has shown that KUS121, one of the KUSs, improved the visual outcome in patients with central retinal artery occlusion^9^. Thus, KUSs are expected to have several possibilities for treatment of various neurodegenerative diseases in the future.

KUSs are considered to prevent cell death by preventing intracellular ATP decrease, but the mechanism of cell protection by KUSs has not been fully elucidated. We have previously shown that the expression of various genes in retinal ganglion cells (RGCs), isolated by fluorescence-activated cell sorting, was altered by KUS treatment in a model of N-methyl-d-aspartate (NMDA)-induced acute retinal ganglion cell death^10^. In the previous study, 255 genes, whose expression varied significantly between groups according to analysis of variance (ANOVA) at a 1% level of significance, were analyzed. The current study aimed to further elucidate the mechanism by expanding the number of genes searched, by changing the significance level to 5%; we focused on *Edn1* and *Ednrb* in this study.

## Results

### Gene ontology/pathway analysis of expression variant genes

Using analysis of variance (ANOVA), 1084 genes (including isoforms) showed significant changes in expression among four conditions; non-treatment (non-treated), vehicle treatment with intravitreous NMDA injection (NMDA-saline), KUS121 treatment with intravitreous NMDA injection (KUS121) and KUS187 treatment with intravitreous NMDA injection (KUS187) (p < 0.05). Of the 387 transcripts upregulated (fold change > 2) in the NMDA-saline group compared with the non-treated group, 181 (130 genes) and 136 transcripts (95 genes) were downregulated (fold change < 0.5) in the KUS121 and KUS187 groups, respectively, relative to the NMDA-saline group. Of the 335 transcripts downregulated in the NMDA-saline group (fold change < 0.5) compared with the non-treated group, 97 (78 genes) and 93 transcripts (74 genes) were upregulated (fold change > 2) in the KUS121 and KUS187 groups, respectively, relative to the NMDA-saline group. Because both KUS121 and KUS187 have neuroprotective effects, we hypothesized that the effects common among KUSs are crucial in neuroprotection.

Gene ontology (GO)^11^ and pathway analysis^12^ was performed to annotate genes that were altered between the conditions into biological ontology and to specify the altered pathways between conditions. First, GO/Pathway analysis was performed on the genes that were downregulated by NMDA injection and upregulated by KUSs independently for KUS121 (78 genes) or KUS187 (74 genes), and found no common GO terms or pathways between KUS121 and KUS187 (Fig. 1b). On the other hand, when the genes that were upregulated by NMDA injection and downregulated by KUSs analyzed independently for KUS121 (130 genes) and KUS187 (95 genes), GO terms common between KUS121 and KUS187 included excretion (Fig. 1c). Genes related to the excretion GO terms are shown in Table 1; among them, we focused on *Edn1* and *Ednrb*, which have been reported to be related to eye diseases^13–16^.

**Table 1:** Genes related to the excretion GO term and upregulated by NMDA injection and downregulated by KUS121 and KUS187 treatment.

Gene symbol	Description
Mdk	Midkine
Mgll	Monoglyceride lipase
Ednrb	Endothelin receptor type B
Edn1	Endothelin-1
Slc8b1	Solute carrier family 8 member B1
Amn	Amnionless
Ptn	Pleiotrophin
Apela	Apelin receptor early endogenous ligand
Tcirg1	T cell, immune regulator 1, ATPase H + Ttansporting V0 subunit A3
Ccn6	Cellular communication network factor 6

### Expression of endothelin-related mRNA in mouse retina and primary RGCs

**Figure 2:**
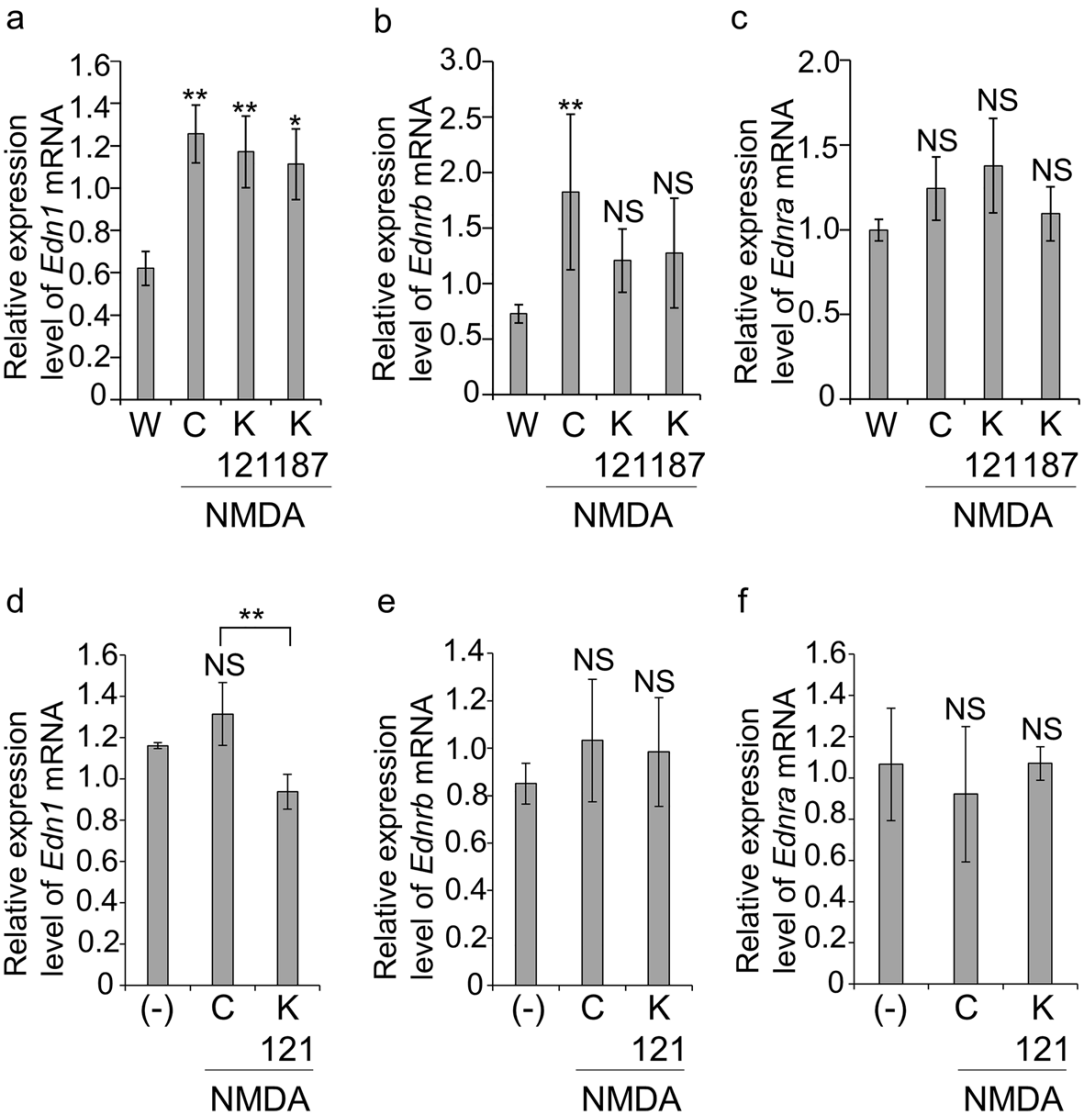
mRNA expressions of endothelin-1 (*Edn1*), endothelin receptor type A (*Ednra*) and endothelin receptor type B (*Ednrb*) in the mouse retina and primary retinal ganglion cells (RGCs). (**a**–**c**) Relative mRNA expression in neural retina. Neural retina of non-treated wild-type mice (W, *n* = 7) and NMDA-injected (C, *n* = 8), NMDA-injected-KUS121-treated (K121, *n* = 8) and NMDA-injected-KUS187-treated (K187, *n* = 8) mice were analyzed. The relative expression levels of *Edn1* (**a**), *Ednrb* (**b**) and *Ednra* (**c**) mRNA were analyzed using qRT-PCR. The ratios of mRNA expression of each gene to that of glyceraldehyde-3-phosphate dehydrogenase were calculated. *p < 0.05 and **p < 0.01, vs. W, Tukey’s honestly significant difference (HSD). *NS* no significant difference compared with W, Tukey’s HSD. (**d**–**f**) Relative mRNA expression in primary RGCs. Primary RGCs isolated from 3-day-old rats by two-step immunopanning were cultured with or without KUS121 (50 µM) for 2 h and then with or without KUS121 and with or without NMDA (500 µM) for another 4 h. The relative expression levels of *Edn1* (**d**), *Ednrb* (**e**) and *Ednra* (**f**) mRNA were analyzed using qRT-PCR. (-): without KUS121 without NMDA *n* = 3, C: with NMDA without KUS121, *n* = 3, K121: with NMDA with KUS121, *n* = 3, respectively. The ratios of mRNA expression of each gene to that of glyceraldehyde-3-phosphate dehydrogenase were calculated. **p < 0.01, control vs. KUS121, *NS* no significant difference compared with (-), Tukey’s HSD.

To confirm the change of expression in the retina, the mRNA levels of *Edn1* and *Ednrb* were analyzed using qRT-PCR. Thereafter, the neural retina of wild-type non-treated (*n* = 7), NMDA-injected, NMDA-injected KUS121-treated and NMDA-injected KUS187-treated mice (*n* = 8, each) were analyzed. The mRNA expression of *Edn1* was significantly higher in all three NMDA-injected groups than in the non-treated group [*p* = 0.001, *p* = 0.003 and *p* = 0.016, respectively; Tukey’s honestly significant difference (HSD)] (Fig. 2a). On the other hand, *Ednrb* mRNA expression was significantly upregulated in the NMDA-injected control group compared with the wild-type non-treated group (*p* = 0.005; Tukey’s HSD). However, there was no significant difference between the NMDA-injected KUS121-treated and NMDA-injected KUS187-treated groups relative to the non-treated group (Fig. 2b). *Ednra,* which encodes another endothelin receptor subtype, showed no significant difference between any of the groups (Fig. 2c).

Next, we examined gene expression in primary RGCs. We first confirmed that the live cell numbers decreased and increased following NMDA and KUS121 administration, respectively. However, the changes were not statistically significant (Supplementary Fig. S1). The expression of *Edn1* mRNA was upregulated by NMDA administration and downregulated by KUS121 administration (non-stress (-) vs. glucose-free (control); *p* = 0.078, control vs. KUS; *p* = 0.009, respectively; Tukey’s HSD; Fig. 2d). *Ednrb* mRNA tended to be upregulated by NMDA and downregulated by KUS121, although not statistically significantly (non-stress (-) vs. glucose-free (control); *p* = 0.56, control vs. KUS; *p* = 0.96, respectively; Tukey’s HSD, Fig. 2e). In contrast, *Ednra* exhibited the opposite trend and was downregulated by NMDA and upregulated by KUS121, although not statistically significantly (non-stress (-) vs. glucose-free (control); *p* = 0.77, control vs. KUS; *p* = 0.77, respectively, Tukey’s HSD, Fig. 2f).

### Expression of EDN1 and EDNRB proteins in the mouse retina

**Figure 3:**
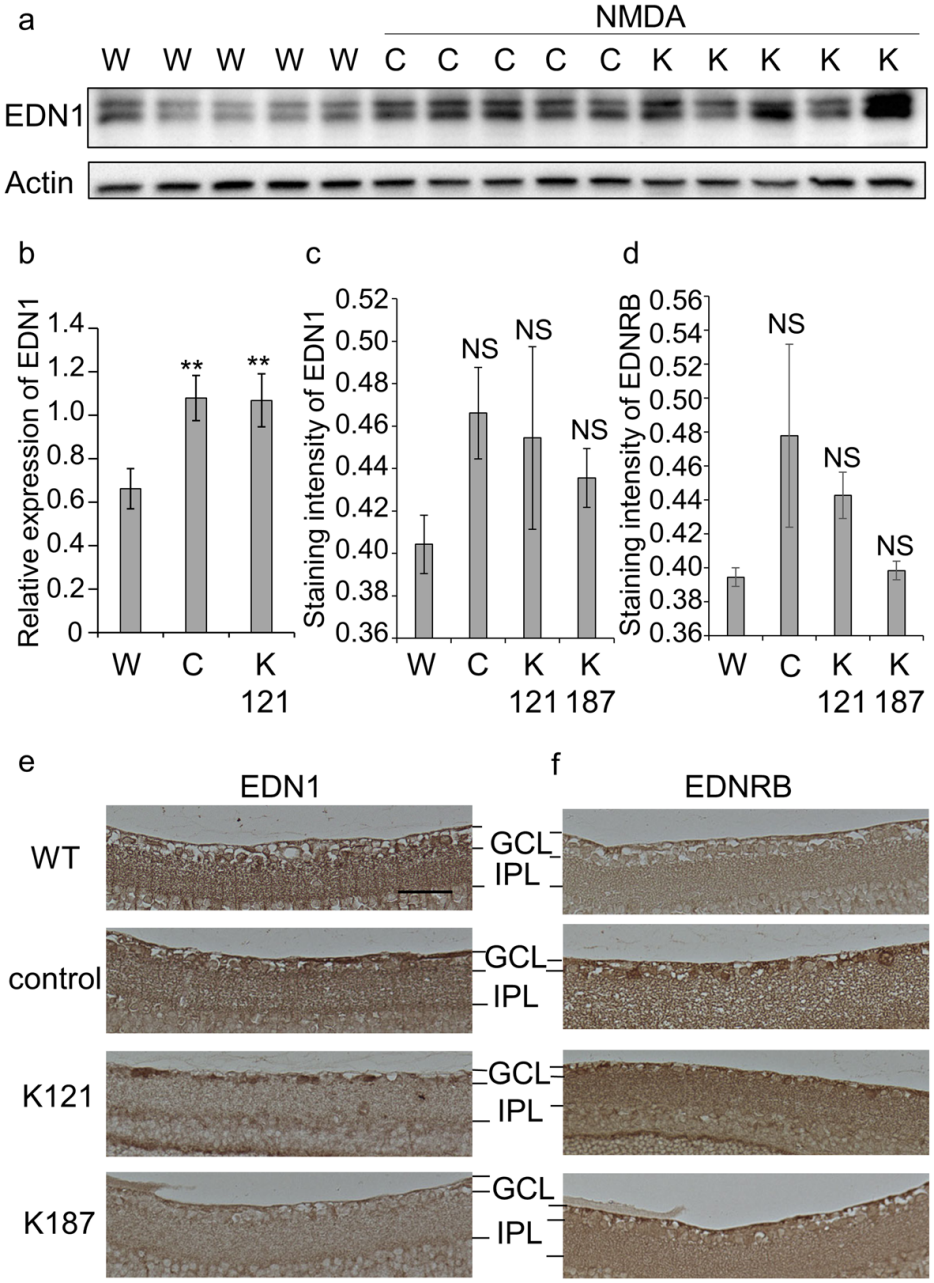
Protein expression of endothelin-1 (EDN1) and endothelin receptor type B (EDNRB) in retinal tissue. (**a**) The retina of non-treated wild-type (labeled ‘‘W’’), NMDA-injected (control, labeled ‘‘C’’), or NMDA-injected-KUS121-treated mice (labeled ‘‘K’’) was analyzed by western blotting using an anti-EDN1 antibody. Actin was used as a loading control. Complete scans of western blots are shown in Supplementary Fig. S2a. (**b**) Comparison of EDN1 expression shown as ratios to actin (*n* = 5, for all treatments). **p < 0.01, vs. W, Tukey’s HSD. (**c**–**f**) Vertical sections of non-treated wild-type, NMDA-injected, NMDA-injected-KUS121-treated and NMDA-injected-KUS187-treated mice retinae were stained with an anti-EDN1 antibody or anti-EDNRB antibody. (**c**,**d**) Staining intensities of RGC layers with anti-EDN1 (**c**) or anti-EDNRB (**d**) antibody. The staining intensity of the RGC layer at distances of 400–800 μm from the optic nerve head was analyzed using BZ II Analyzer software. W: non-treated wild-type, C: NMDA-injected, K121: NMDA-injected-KUS121-treated, K187: NMDA-injected-KUS187-treated. (*n* = 3, for C and K121, *n* = 2, for W and K187) *NS* no significant difference compared with W, Tukey’s HSD. (**e,f**) Vertical sections of non-treated wild-type (WT), NMDA-injected (control), NMDA-injected-KUS121-treated (K121) and NMDA-injected-KUS187-treated mice (K187) retinae. The black bar represents 100 µm. *GCL* ganglion cell layer, *IPL* inner plexiform layer.

Protein expression in the neural retina was examined by western blotting. The retinas of wild-type non-treated, NMDA-injected and NMDA-injected KUS121-treated mice (*n* = 5 per treatment) were used. There was a significant increase in EDN1 expression in NMDA-injected and NMDA-injected KUS121-treated mice retinae than in non-treated mice retinae (*p* = 0.007 and *p* = 0.008, respectively; Tukey’s HSD; Fig. 3a,b, Supplementary Fig. S2a). However, there was no apparent difference in EDNRB expression between the groups in western blotting. Immunohistochemical analysis showed that the expression of both the EDN1 and EDNRB proteins appeared to be higher in the retinal ganglion cells of the NMDA-injected mice retinae than in those of wild-type non-treated mice and had lower expression in the retinae of NMDA-injected KUS-treated mice than in the retinae of NMDA-injected mice. However, the changes were not significant (Fig. 3c–f).

### Influence of the endothelin receptor signaling on the protective effect of KUS121 in cultured cells under stress conditions

As a next step, the possible involvement of the endothelin receptor signaling in the protective effect of KUS121 was investigated using 661W retinal cells, a retinal cell line derived from mouse retinal tumors and reported to be a retinal ganglion precursor-like cell line^17,18^.

Hereafter, we focused on KUS121, which has been more widely studied than KUS187^6–8^ and evaluated in a clinical trial^9^.

**Figure 4:**
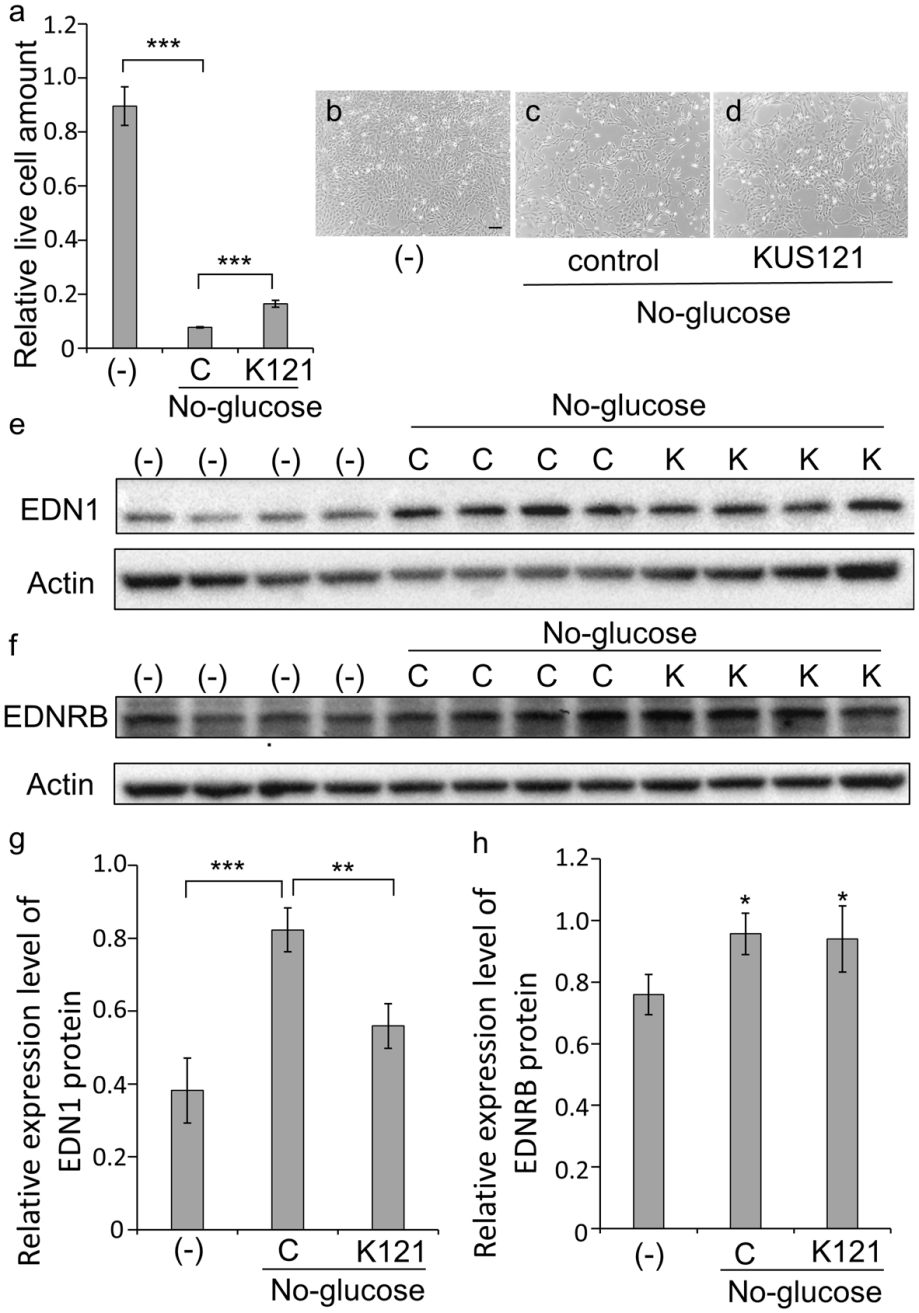
EDN1 and EDNRB protein expression and cell viability in 661W cultured cells under glucose-free conditions. (**a**–**d**) The relative amount of live cells was measured using WST-8 following 48 h treatment with DMEM/high glucose media or DMEM/glucose-free media with or without KUS121 (100 µM). (**a**) Quantitative analysis of live cells (*n* = 3, for all treatments). 661W cells were cultured with high glucose [labeled ‘‘(-)’’] or treated in glucose-free media with (labeled ‘‘K121’’) or without (control, labeled ‘‘C’’) KUS121. (**b**–**d**) Representative images of 661W cells cultured under each condition. The black bar represents 50 µm. (**e**–**h**) Protein expression of EDN1 (**e**,**g**) and EDNRB (**f**,**h**) in 661W cells was analyzed by western blotting. 661W cells were cultured with high glucose media [labeled ‘‘(-)’’] or with glucose-free media with (labeled ‘‘K’’) or without (control, labeled ‘‘C’’) KUS121 for 24 h before the analysis. Actin was used as a loading control. Complete scans of western blots are shown in Supplementary Fig. S2b,c. Relative expression of EDN1 (**g**) and EDNRB (**h**) was shown as a ratio to actin (*n* = 4, for both treatments). *p < 0.05, **p < 0.01 and ***p < 0.001, compared with the control (in **a** and **g**) or to the cells cultured in high glucose media (in **h**), Tukey’s HSD.

When 661W cells were cultured under glucose-free conditions, cell death occurred within 48 h; however, KUS121 attenuated cell death [Fig. 4a–d, non-stress (-) vs. glucose-free (control); *p* < 0.001, control vs. KUS; *p* < 0.001, Tukey’s HSD].

Following confirmation of EDN1 and EDNRB protein expression in 661W cells (Fig. 4e,f, Supplementary Fig. S2b,c), we evaluated the expression changes when cells were cultured for 24 h under glucose-free conditions. The protein expression of EDN1 was upregulated under glucose-free conditions, whereas KUS121 suppressed EDN1 upregulation [Fig. 4e,g, (-) vs. control; *p* < 0.0001, control vs. KUS; *p* = 0.001, Tukey’s HSD]. Similarly, EDNRB protein expression was also upregulated under glucose-free conditions (Fig. 4f,h). Afterward, to evaluate the possible influence of the endothelin receptor signaling on the protective effect of KUS, we examined whether an antagonist or agonist of endothelin influences the suppression of cell death by KUS121 under glucose-free conditions in 661W cells.

**Figure 5:**
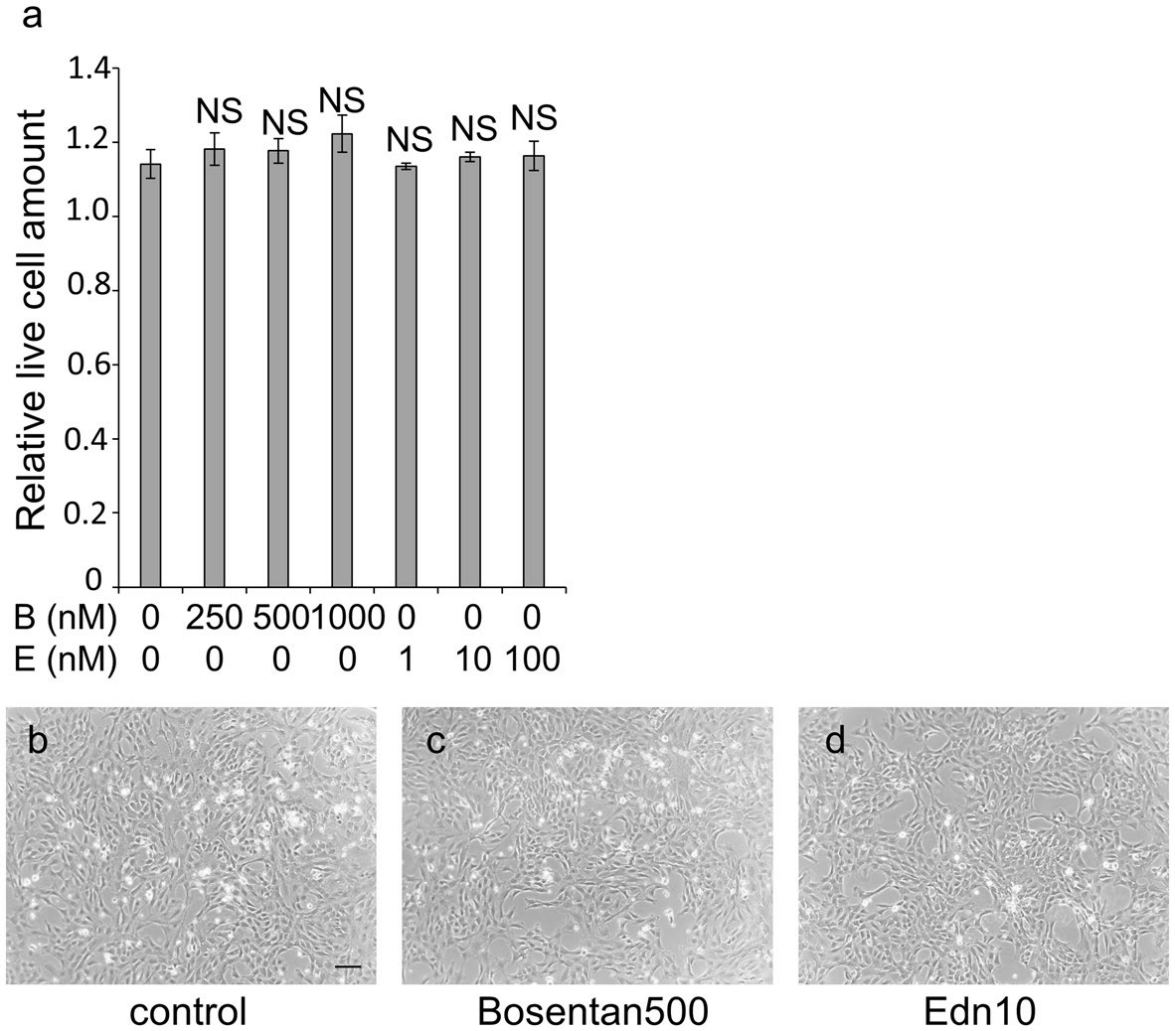
Cell viability of 661W cells cultured under stress-free conditions with an endothelin antagonist or agonist. The relative amount of live cells was measured using WST-8 following 48 h treatment with or without bosentan (250, 500 or 1000 nM) and with or without endothelin-1 (1, 10 or 100 nM). (**a**) Quantitative analysis of live cells (*n* = 3, for all treatments). *NS*: no significant difference vs. without bosentan nor endothelin-1, Tukey’s HSD. *B*: bosentan, *E*: endothelin-1. (**b**–**d**) Representative images of 661W cells cultured under each condition. Bar: 50 µm.

**Figure 6:**
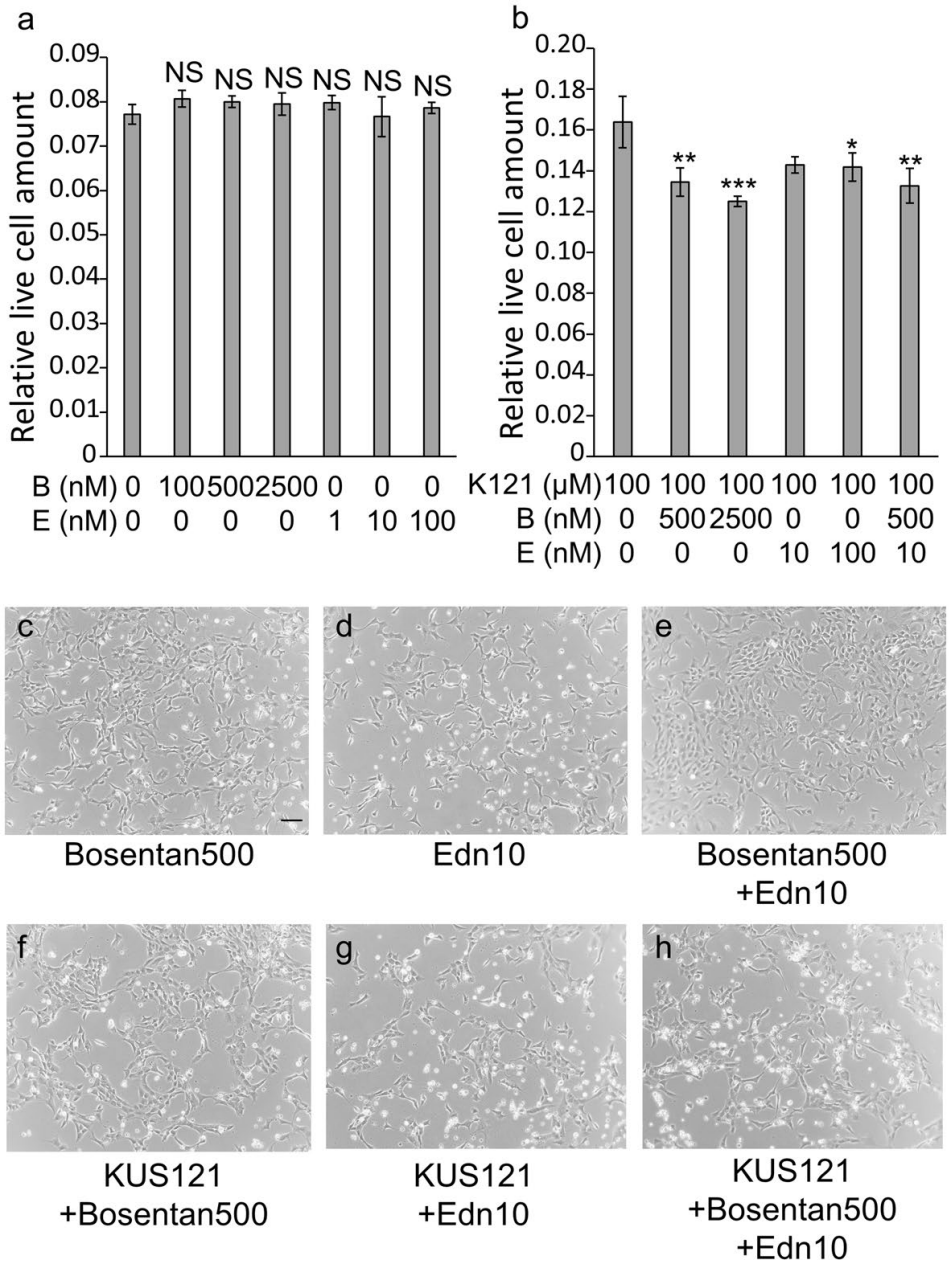
Cell viability of 661W cells cultured under glucose-free conditions with an endothelin antagonist or agonist. The amount of live cells was measured using WST-8 following 48 h treatment in glucose-free media with or without KUS121 (100 µM), with or without bosentan (100; 500; 2500 nM), and with or without endothelin-1 (1; 10; 100 nM). (**a**,**b**) Quantitative analysis of live cells (*n* = 3, for all treatments). 661W cells were cultured under glucose-free conditions, with (**b**) or without KUS (**a**), with or without bosentan (100; 500; 2500 nM), and with or without endothelin-1 (1; 10; 100 nM). *NS*: no significant difference, vs. glucose-free without bosentan nor endothelin-1, Tukey’s HSD. *p < 0.05, **p < 0.01, ***p < 0.001, vs. no glucose with KUS, Tukey’s HSD. K121: KUS121, *B*: bosentan, *E*: endothelin-1. (**c**–**h**) Representative images of 661W cells cultured under each condition. Bar: 50 µm.

Bosentan, an endothelin antagonist and endothelin-1 did not show cytoprotective or cytotoxic effects under stress-free conditions (Fig. 5a–d). When bosentan and/or endothelin-1, was added to cells under glucose-free conditions and cultured for 48 h, the amount of live cells did not change; bosentan and endothelin-1 also showed no cytotoxicity to 661W cells under glucose-free conditions (Fig. 6a,c–e). Thereafter, bosentan and/or endothelin-1 were added to KUS121-treated 661W cells and cultured under glucose-free conditions for 48 h. The amount of live cells was significantly smaller when KUS121 was added together with bosentan than without (KUS121 vs. KUS121 + bosentan 500 nM: *p* = 0.005, KUS121 vs. KUS121 + bosentan 2500 nM: *p* < 0.001, Tukey’s HSD, Fig. 6b,f). Similarly, when endothelin-1 was added to KUS121-treated 661W cells, the amount of live cells was significantly smaller than those supplemented with KUS121 alone (KUS121 vs. KUS121 + endothelin-1 10 nM: *p* = 0.050, KUS121 vs. KUS121 + endothelin-1 100 nM: *p* = 0.038, Tukey’s HSD, Fig. 6b,g). When both endothelin-1 (10 nM) and bosentan (500 nM) were added to 661W cells with KUS121, the amount of live cells was significantly smaller than when KUS121 was added alone (*p* = 0.003, Tukey’s HSD, Fig. 6b,h).

**Figure 7:**
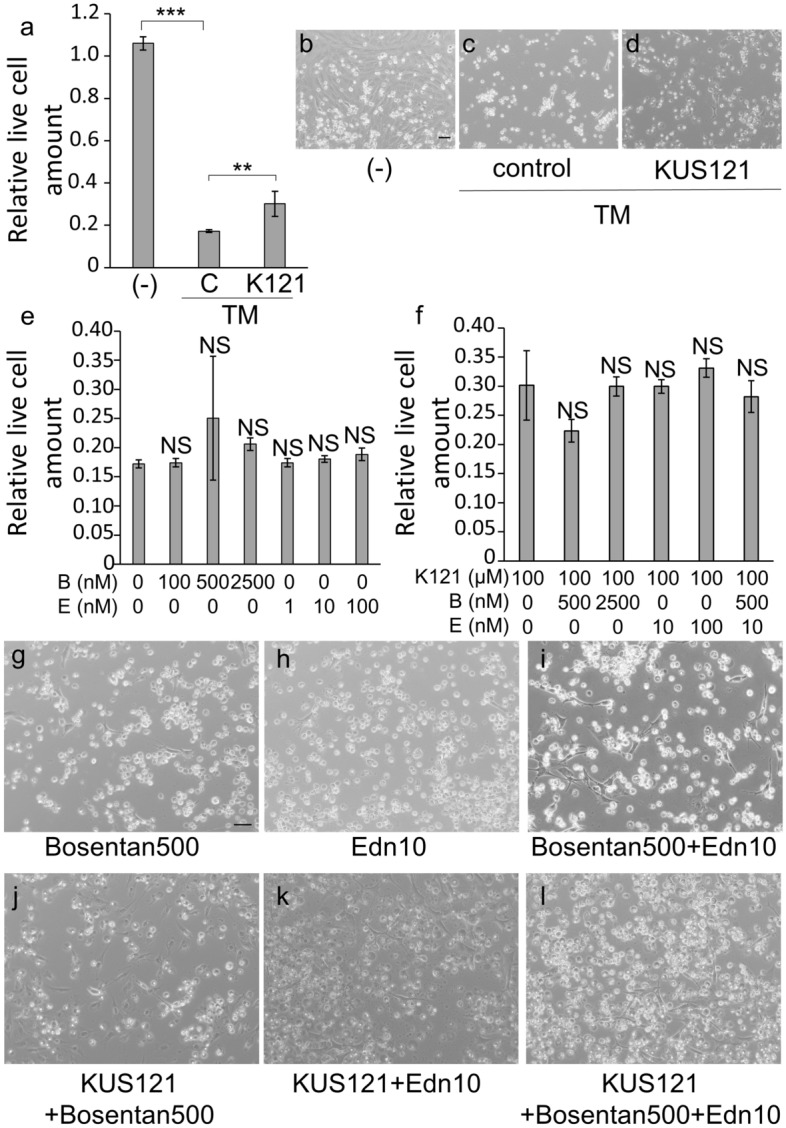
Cell viability of 661W cells under endoplasmic reticulum stress. Cells were analyzed following 40 h treatment with tunicamycin (TM, 0.2 µg/mL) and with or without KUS121 (100 µM). (**a**) Quantitative analysis of live cells (*n* = 3, for all treatments). 661W cells were treated without TM and without KUS121 [labeled ‘‘(-)’’], with TM and without KUS121 (control, labeled ‘‘C’’), or with TM and KUS121 (labeled ‘‘K121’’). **p < 0.01, ***p < 0.001, Tukey’s HSD. (**b**–**d**) Representative images of 661W cells cultured under each condition. (**e**,**f**) Quantitative analysis of live cells (*n* = 3, for all treatments). 661W cells were treated with TM and with (**f**) or without KUS121 (**e**) with bosentan (0; 100; 500; 2500 nM) or endothelin-1 (0; 1; 10; 100 nM). *NS* no significant difference, vs. control (without bosentan nor endothelin-1) in (**e**), vs. KUS121 (without bosentan nor endothelin-1) in (**f**), Tukey’s HSD. K121: KUS121, *B*: bosentan, *E*: endothelin-1. (**g**–**l**) Representative images of 661W cells cultured under each condition. Bar: 50 µm.

Then, the involvement of the endothelin receptor signaling in the protective effect of KUS under tunicamycin (TM)-induced endoplasmic reticulum (ER) stress was investigated. The addition of TM significantly reduced the amount of live cells (*p* < 0.001, Tukey’s HSD, Fig. 7a–c). The amount of live cells was significantly higher when KUS121 was added under TM-induced ER stress for 40 h (*p* = 0.004, Tukey’s HSD, Fig. 7a,c–d). When bosentan and/or endothelin-1 were added to the cells and cultured under TM-induced ER stress for 40 h, the amount of live cells did not change; bosentan and endothelin-1 showed no cytotoxicity to 661W cells (control vs. all other groups: *p* > 0.05, Tukey’s HSD, Fig. 7e,g–i). Under TM-induced ER stress, the amount of live cells did not significantly differ among cells treated with KUS121 alone and cells treated with KUS121 together with bosentan and/or endothelin-1 (KUS121 vs. all other groups: *p* > 0.05, Tukey’s HSD, Fig. 7f,j–l).

**Figure 8:**
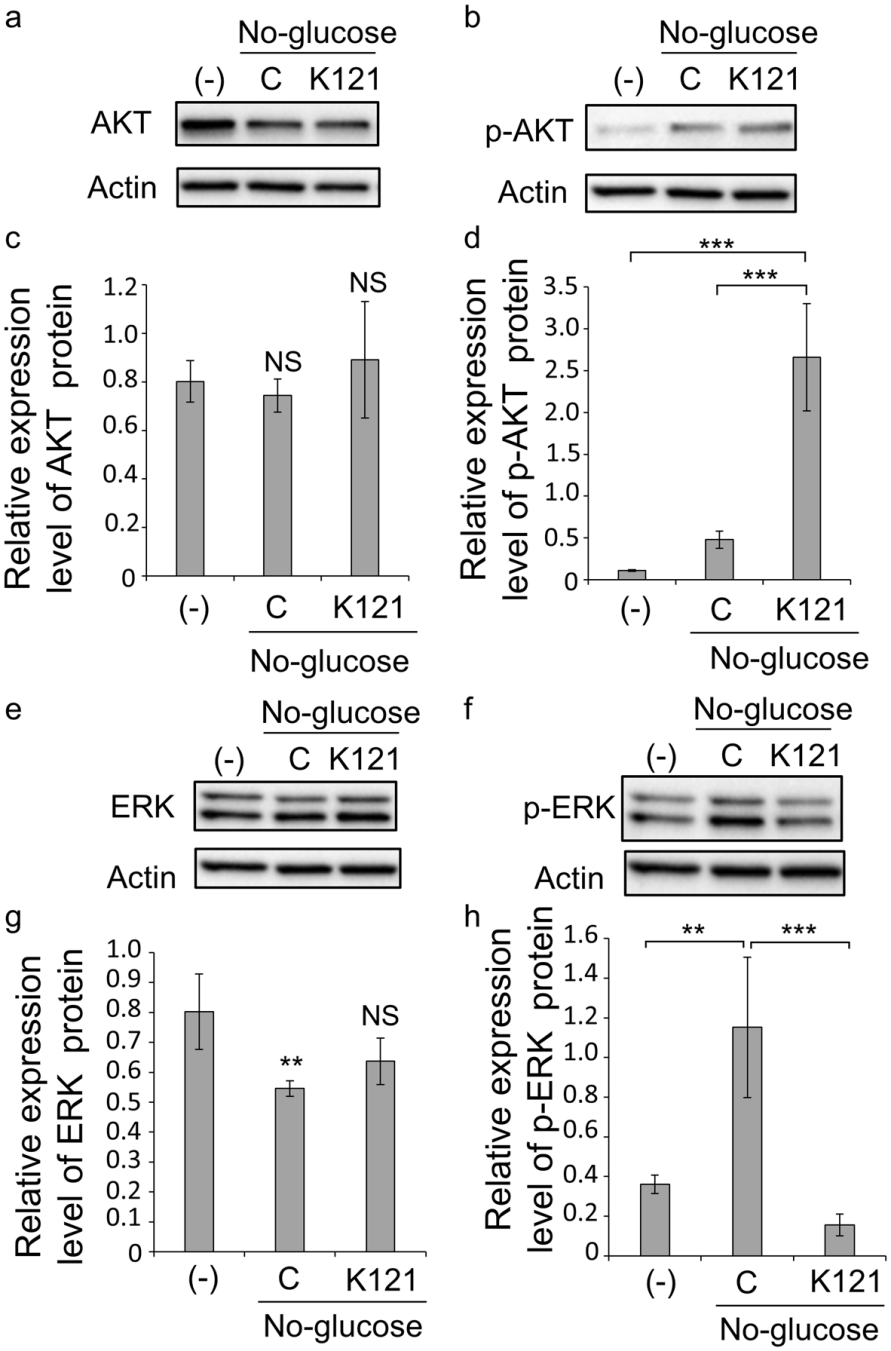
AKT, p-AKT, ERK and p-ERK protein expression in 661W cultured cells under glucose-free conditions. (**a**–**d**) Protein expression of AKT (**a,c**) and p-AKT (**b,d**) in 661W cells was analyzed by western blotting. Actin was used as a loading control. Complete scans of western blots are shown in Supplementary Fig. S3a,b. (-): cultured in high-glucose media, C: cultured with glucose-free media without KUS121, K121: cultured with glucose-free media with KUS121 (100 µM). Relative expression of AKT(**c**) and p-AKT (**d**) is shown as a ratio to actin (*n* = 4, for both treatments). NS: no significant difference compared with (-) (in **c**), ***p < 0.001, compared with cells cultured in glucose-free media with KUS121 (in **d**), Tukey’s HSD. (**e**–**h**) Protein expression of ERK (**e**,**g**) and p-ERK (**f**,**h**) in 661W cells was analyzed by western blotting. Actin was used as a loading control. Complete scans of western blots are shown in Supplementary Fig. S4a,b. (-): cultured in high-glucose media, C: cultured with glucose-free media without KUS121, K121: cultured with glucose-free media with KUS121 (100 µM). Relative expression of ERK (**g**) and p-ERK (**h**) is shown as a ratio to actin (*n* = 4, for both treatments). **p < 0.01, NS: no significant difference compared with (-) (in **g**), **p < 0.01, ***p < 0.001, compared with cells cultured in glucose-free media without KUS121 (in **h**), Tukey’s HSD.

We next examined how KUS affects the phosphorylation of *Akt* and *Erk*, which are downstream genes of *Ednrb*. Western blotting of 661W cells revealed that phosphorylation of AKT was markedly enhanced under glucose-free conditions and further enhanced by KUS121 administration (*p* < 0.001, both non-stress (-) vs. KUS and glucose-free (control) vs. KUS, Tukey’s HSD, Fig. 8a–d, Supplementary Fig. S3). In contrast, the phosphorylation of ERK was promoted under glucose-free conditions and suppressed by KUS121 treatment (*p* = 0.001, non-stress (-) vs. glucose-free (control), *p* < 0.001, glucose-free (control) vs. KUS, Tukey’s HSD, Fig. 8e–h, Supplementary Fig. S4).

To examine whether KUS modulates endothelin signaling, we performed an experiment using ready-to-use CHO cells, which express Ednrb on the cell surface and whose fluorescence is excited by its calcium response. Fluorescence intensity was measured after 16 h of incubation under glucose-free conditions with or without KUS. Although the results did not significantly differ, the fluorescence intensity increased under glucose-free conditions and decreased following the addition of KUS (Supplementary Fig. S5).

## Discussion

In the present study, to elucidate the underlying mechanisms of neuroprotection by KUSs, we focused on two genes, *Edn1* and *Ednrb*, and investigated whether these genes are involved in the protective effects of KUSs.

Endothelin-1, originally isolated from vascular endothelial culture supernatants and identified as a potent vasoconstrictor, is expressed in the lungs and other body organs and its secretion is known to be enhanced by various stresses. Endothelin receptor antagonists are currently being used clinically in pulmonary arterial hypertension and as ameliorative agents for vascular disorders in systemic scleroderma^19,20^.

In relation to eye diseases, *Edn1* has been reported to be associated with diabetic retinopathy^21^. Moreover, the polymorphism of its receptor, *Ednra*, in leukocyte DNA has been found to be associated with visual field severity in patients with normal-tension glaucoma^22^.

It has been also reported that *Ednrb* is upregulated in RGCs in an ocular hypertension rat model and RGC reduction is less in an *Ednrb*-knockout model^15^.

Additionally, it has been reported that 8 days of oral bosentan administration, an endothelin receptor antagonist, decreased ocular perfusion pressure in glaucoma patients and normal individuals^23^.

Using mouse retinae, qRT-PCR revealed that both *Edn1* and *Ednrb* were increased by NMDA, as expected and *Ednrb* was decreased by KUS administration. Moreover, western blotting analysis also showed an increase in EDN1 expression in NMDA intravitreous-injected retinas. The failure to clearly reproduce the decreased expression by KUS in EDN1 and EDNRB was likely because the samples used for RT-PCR and western blotting comprised whole neural retinae while RGCs account for less than a few percentages of the total cells in whole neural retinae, making it difficult to reflect the changes in RGCs. Immunostaining showed that EDN1 and EDNRB were expressed more strongly in the RGC layer of NMDA-injected mice retinae and slightly weakened by KUSs treatment. Considering that the expression level of *Edn1* and its receptors may vary in vivo and in vitro, we conducted experiments using primary RGCs and confirmed that the expression of *Edn1* and *Ednrb* mRNAs increased following NMDA administration and decreased following KUS administration.

We then questioned whether the endothelin signals through the endothelin receptors mediated the protective effect of KUS. In other words, the question is whether the suppressed endothelin signals by KUSs results in or results from cytoprotection.

To examine this, we conducted experiments using 661W retinal cells. We confirmed that EDN1 expression increased under glucose-free conditions and decreased with the addition of KUS in 661W cells. We then added an endothelin receptor antagonist, bosentan, or endothelin-1 to 661W cells cultured under stress and observed that the addition of bosentan to KUS121-treated cells significantly reduced the cell viability compared with that in cells treated with KUS121 only. This result shows that the protective effect of KUS121 is partially attenuated by the addition of bosentan, suggesting the involvement of the endothelin receptor in the protective effect of KUS. Considering the fact that *Edn1* and *Ednrb* expression is reduced by KUS treatment, this suggests that the protective effect of KUS stems from reducing the endothelin receptor expression and consequently, the functions of these receptors. Similarly, the addition of endothelin-1 to 661W cells with KUS121 significantly reduced the cell viability compared with cells treated with KUS121, and the reduction in cell viability was more remarkable at higher concentrations of endothelin-1. This suggests the possibility that the protective effect of KUS is attenuated when the endothelin signal is enhanced above a certain level. Furthermore, this reinforces the possibility that KUS has a protective effect through suppression of the endothelin receptor signaling.

The above results were obtained under glucose-free conditions, i.e. when energy production was directly suppressed. To examine the effects under stress conditions that do not directly suppress energy production, we conducted similar experiments using tunicamycin, an endoplasmic reticulum stress inducer. Similarly, the addition of KUS121 showed significant protective effects, however, the addition of bosentan or endothelin-1 to KUS-treated cells did not significantly change the cell viability. In other words, under TM-induced ER stress, the involvement of the endothelin receptors in the protective effect of KUS was unclear, suggesting that the endothelin signaling might not have a major influence on cell death under certain conditions.

There are two major downstream pathways of *Ednrb*: the phosphatidylinositol 3 kinase/AKT/eNOS signaling pathway and mitogen-activated protein kinase pathway^24,25^. In this study, we focused on phosphorylation of *Akt* and *Erk* from each pathway, respectively, in 661W cells supplemented with KUS under glucose-free conditions. First, the enhanced phosphorylation of AKT under glucose-free conditions was further enhanced by KUS administration (Fig. 8b,d, Supplementary Fig. S3b). KUS reportedly enhances the phosphorylation of AKT^4^. As *Akt* is involved in signaling from multiple pathways, phosphorylation of AKT with KUS may have occurred through pathways other than those downstream of *Ednrb*. For ERK, unlike AKT, phosphorylation was promoted under glucose-free conditions and suppressed by KUS administration (Fig. 8f,h, Supplementary Fig. S4b). In order to know whether KUS phosphorylates ERK directly or through Ednrb, a functional assay was performed and indicated that Ednrb signaling was decreased by KUS. Although the possibility that KUS directly suppresses ERK phosphorylation cannot be denied, KUS may likely suppress the function of Ednrb by reducing Ednrb expression.

In conclusion, these results suggest that the cytoprotective effects of KUS involve the suppression of endothelin receptor signaling. Although the mechanisms of neuroprotection by KUSs remain to be further elucidated, the information revealed in the current study will be useful in promoting the future clinical application of KUS.

## Methods

### Experimental animals and administration of KUSs

This study was conducted in accordance with the Association for Research in Vision and Ophthalmology (ARVO) Statement for the Use of Animals in Ophthalmic and Vision Research. The study is reported in accordance with ARRIVE guidelines. All protocols were approved by the Institutional Review Board of the Kyoto University Graduate School of Medicine (MedKyo 12245, 13221, 14213, 15531, 16501). B6.Cg-Tg (Thy 1-CFP) 23 Jrs/J mice, which express cyan fluorescent protein (CFP) in RGCs^26^, were obtained from the Jackson Laboratory (Bar Harbor, ME, USA) and were used for RGC collection by FACS as previously reported^10^. Wild-type mice (C57/BL6), which share a genetic background with Thy1-CFP mice, were purchased from Japan SLC Inc (Shizuoka, Japan). Mice were kept under a 14 h light/10 h dark cycle and fed ad libitum. All experiments were conducted in 2–3-month-old male mice to minimize individual variabilities. KUS121 and KUS187 (50 mg/kg/day each) were orally administered daily to Thy1-CFP mice or wild-type mice using a feeding tube and 7 days after the start of medication, a vitreous injection of NMDA (5 nmol) was administered using a 33-gauge needle. The NMDA-saline group mice orally received a vehicle [5% Cremophor EL (Sigma) in phosphate-buffered saline (PBS)], instead of KUSs, for 7 days as a control. Before intravitreous NMDA injection, the mice were anesthetized by intraperitoneal injection of pentobarbital (50 mg/kg) and mydriasis with tropicamide and phenylephrine eye drops (0.5% each)^10^. The retinas were collected unilaterally from each mouse and analyzed.

### Analysis of the RNA-sequencing results

The results of RNA-sequencing, which have been reported previously^10^, were mapped to a reference sequence (mouse mm10, USCS genome browser), after which the expression levels were calculated and the expression value normalized by quantile normalization methods as previously reported^10^. Expression changes among conditions were analyzed using ANOVA. Using fold change analysis, genes whose expression differed by more than two fold (indicated as fold change > 2) or less than 0.5-fold (indicated as fold change < 0.5) were selected and further studied. Using Metascape (https://metascape.org/gp/index.html#/main/step1), pathway analysis and GO analysis^11^ were performed on genes upregulated in the NMDA-saline group and downregulated in the KUS121 or KUS187 group (130 and 95 genes, respectively) and those downregulated in the NMDA-saline group and upregulated in the KUS121 or KUS187 group (78 and 74 genes, respectively).

### Quantitative reverse transcription-polymerase chain reaction (RT-PCR) of mice neural retinae

Mice eyeballs were enucleated 4 h after intravitreous injection of NMDA under pentobarbital overdose and immersed in cold Hanks’ balanced salt solution. The sclera was peeled through incisions made using pinholes in the corneas to remove the mixture of retinal pigment epithelium, choroid and sclera from the neural retina, as previously described^10^. The lens and iris were removed.

RNA was extracted from the neural retina using RNeasy Mini Kit (Qiagen, Venlo, Netherlands). The mRNA was reverse-transcribed using SuperScript™ III First-Strand Synthesis SuperMix for qRT-PCR (Invitrogen, Waltham, MA, USA). Thereafter, complementary DNA was amplified by PCR (Stratagene MX3000P QPCR System; Agilent Technologies, Santa Clara, CA, USA) using TB Green Premix ExTaq (Takara Bio Inc., Shiga, Japan) at an annealing temperature of 60 °C. Each eye was analyzed separately. The primers used were as follows: *Edn1*: primer-F, GCCCAAAGTACCATGCAGAA; primer-R, GATGCCTTGATGCTATTGCTGA; *Ednrb*: primer-F: CATGCGCAATGGTCCCAATA, primer-R: GCTCCAAATGGCCAGTCCTC, *Ednra*: primer-F: CATCGGCATTAACCTGGCAAC, primer-R: GGACTGGTGACAACAGCAACAGA. Glyceraldehyde-3-phosphate dehydrogenase was used as an internal standard.

### Western blotting with mice neural retinae and 661W cells

Mouse neural retinae were prepared as described in the preceding section. The 661W cells were cultured under glucose-free conditions with or without KUS for 24 h. Mouse neural retinae and 661W cells were lysed using lysis buffer [RIPA Buffer (25 mM Tris–HCl (pH 7.6), 150 mM NaCl, 1% NP-40, 1% sodium deoxycholate and 0.1% sodium dodecyl sulfate], inhibitor cocktail [1.04 mM 4-(2-Aminoethyl) benzenesulfonyl fluoride hydrochloride, 0.8 µM aprotinin, 20 µM leupeptin and 14 µM E64], 0.5 mM dithiothreitol, 1 mM ethylenediaminetetraacetic acid, 0.10% Chaps, 5 mM β-glycerophosphate, 1 mM NaF, 1 mM NaVaO_4_, 1 mM Nappi and 0.5 mM phenylmethylsulfonyl fluoride. Protein concentration was determined using bicinchoninic acid and 5 µg (for retina), 7.5 µg (for cells for EDN1 and EDNRB) and 2.1 µg (for cells for AKT, p-AKT, ERK, p-ERK) of protein were used for western blot experiments. The primary antibodies used to probe the blots were mouse anti-EDN1 (1:400; Abcam, Cambridge, UK), rabbit anti-EDNRB (1:400; Alomone Labs, Jerusalem, Israel), rabbit anti-AKT (1:1000, Cell Signaling Technology, Danvers, MA, USA), rabbit anti-phospho-AKT (1:2000, Cell Signaling Technology), rabbit anti-ERK (1:20,000, Sigma-Aldrich, St. Louis, MO, USA), rabbit anti-phospho-ERK (1:1000, Cell Signaling Technology) and anti-actin (1:5000; Sigma-Aldrich). The secondary antibodies used were HRP-conjugated anti-mouse IgG (1:5000) and HRP-conjugated anti-rabbit IgG (1:5000). Actin was used as the loading control.

### Immunohistological evaluation of retinas

Wild-type non-treated eyeballs or eyeballs after 4 h of NMDA injection in mice treated with KUS121, KUS187, or vehicle (saline, control) were enucleated after pentobarbital overdose. A marking dye (Davidson Marking System; Bradley Products, Inc., Bloomington, MN, USA) was placed on the edge of the superior conjunctiva to identify the superior portion of the retina, as previously described^10^. The eyes were fixed in 4% paraformaldehyde for 24 h at 4 °C and embedded in paraffin. Serial 6-mm paraffin-embedded sections passing through the center of the optic nerve head were selected. Before staining, antigen retrieval was performed using Tris–EDTA buffer (pH 9.0) at 95 °C for 20 min. The primary antibodies were mouse anti-EDN1 antibody (1:200; Abcam) and rabbit anti-EDNRB antibody (1:100; Alomone Labs) and the secondary antibodies were biotinylated anti-mouse IgG (1:250; Vector Laboratories, Burlingame, CA, USA) for EDN1 and biotinylated anti-mouse IgG (1:250; Vector Laboratories) for EDNRB. The sections were stained with a Peroxidase Stain DAB Kit (Nacalai Tesque, Kyoto, Japan) and imaged under an optical microscope (BZ-9000; Keyence, Osaka, Japan). The staining intensity of the RGC layer within 400–800 µm from the edge of the optic nerve head was analyzed using BZ II Analyzer software (Keyence).

### Culture of 661W cells

Cells were treated with DMEM/high glucose (4.5 g/L) media containing 0.1% fetal bovine serum, DMEM/glucose-free media (09891-25; Nacalai Tesque) containing 0.1% fetal bovine serum, or DMEM/high glucose media containing 0.1% fetal bovine serum with tunicamycin (TM) (0.2 µg/mL, 35638-74; Nacalai Tesque). To assess cell viability, cells were cultured in the aforementioned medium with or without KUS121 (100 µM) and with bosentan (0; 100; 500; 2500 nM; MedChemExpress LLC, Monmouth Junction, NJ, USA), and/or endothelin-1 (0, 1, 10, 100 nM; Peptide, Osaka, Japan) and incubated at 37 °C for 40 h (under TM-stress conditions) or 48 h (under glucose-free conditions). In experiments involving 661W cells, KUS was added simultaneously with the stressor.

For cell viability assays, cells were washed using Dulbecco’s phosphate-buffered saline and incubated with Cell Count Reagent SF (07553-15; Nacalai Tesque) for 20 min. Using a Nivo Microplate reader (PerkinElmer, Waltham, MA, USA), the relative amount of live cells was subsequently determined by measuring the absorbance at 450 nm of the formazan produced by WST-8 reduction via intracellular dehydrogenase.

### Preparation, culture and RT-PCR of primary RGCs

Three-day-old rat eyeballs were removed following pentobarbital overdose and immersed in cold Earle’s balanced salt solution. The neural retina was prepared as described for the mouse neural retina. The neural retina was incubated in papain and then RGCs were isolated by two-step immunopanning using rabbit anti-rat macrophage serum (Accurate Chemical & Scientific Corporation, Carle Place, NY, USA) and anti-Thy1 antibodies (culture supernatant of T11D7e2 cells)^27,28^. Isolated cells were cultured in a medium based on a neurobasal medium (Thermo Fisher Scientific, Waltham, MA, USA) containing B27 supplement (Gibco, Grand Island, NY, USA) and were analyzed within 8 days after isolation. Live cell numbers were counted with a TC20 cell counter (Bio RAD, Hercules, CA, USA) after culture in medium with or without NMDA (500 µM) and with or without KUS121 (50 µM) for 24 h.

For RT-PCR, the cells were cultured with or without KUS121 (50 µM) for 2 h and then cultured in a medium with or without KUS121 and with or without NMDA (500 µM) for 4 h before collecting the cells. RNA was extracted from the cells using an RNeasy Micro Kit (Qiagen). cDNA synthesis and RT-PCR were performed as described for the mouse retina. The following primers were used: *Edn1*: primer-F, GACCAGCGTCCTTGTTCCAA; primer-R, TTGCTACCAGCGGATGCAA. *Ednrb*: primer-F, TGGCCATTTGGAGCTGAGAT; primer-R, TCCAAGAAGCAACAGCTCGAT, *Ednra*: primer-F, CTCAACGCCACGACCAAGTT; primer-R, GCAAGCTCCCATTCCTTCTG. Glyceraldehyde-3-phosphate dehydrogenase was used as an internal standard.

### Functional assay

Ready-to-Assay™ ETB Endothelin Receptor Frozen Cells (Merck, Whitehouse Station, NJ, USA), which are CHO cells expressing Ednrb on the cell surface whose fluorescence is excited by its calcium response, were used to examine whether KUS modulates Ednrb function. Cells were cultured in a glucose-free medium with or without KUS121 (50 µM) for 16 h and then Fluo8-NW (AAD Bioquest, Sunnyvale, CA, USA) was added and the fluorescence intensity was measured 5 min later with a Nivo Microplate reader (PerkinElmer).

### Statistical analysis

Data are presented as mean ± standard deviation. A Tukey’s HSD test was used to compare parameters with multiple conditions. Statistical evaluations were performed using commercially available software (SPSS software version 24; IBM Corp., Armonk, NY, USA). The level of statistical significance was set at p < 0.05.

## Supplementary Information

Supplementary Figures.

## Data Availability

All data generated or analyzed during this study are included in this published article and its Supplementary Information files.
