# Supplementary material for: Involvement of endothelins in neuroprotection of valosin-containing protein modulators against retinal ganglion cell damage
Source: Sci Rep. 2022 Sep 28;12:16156. doi: 10.1038/s41598-022-20497-w (PMC9519977; doi:10.1038/s41598-022-20497-w)
Supplement: Supplementary file 1 — Supplementary Figures. [file 41598_2022_20497_MOESM1_ESM.docx]

**Supplementary information**

**Involvement of endothelins in neuroprotection of valosin-containing protein modulators against retinal ganglion cell damage.**

Mami Kusaka, Tomoko Hasegawa, Hanako Ohashi Ikeda, Yumi Inoue, Sachiko Iwai, Kei Iida, and Akitaka Tsujikawa

**
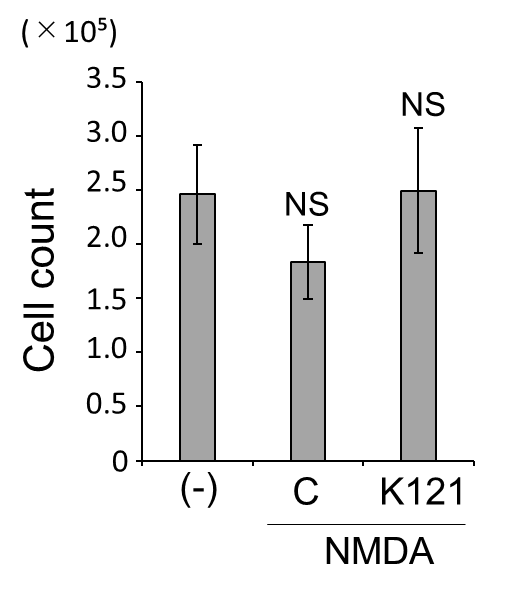
**

**Supplementary Figure S1. Relative live cell numbers in primary RGCs following NMDA and KUS administration.** Primary RGCs isolated from three-day-old rats by two-step immunopanning were cultured with or without KUS121 (50 µM) and with or without NMDA (500 µM) for 24 h. *n* = 4, each. (-): without NMDA without KUS121, C: with NMDA without KUS121, K121: with NMDA and KUS121. NS: no significant difference compared to (-), Tukey’s honestly significant difference (HSD). Abbreviations: KUS, Kyoto University Substances; NMDA, N-methyl-D-aspartate; RGCs, retinal ganglion cells.

**
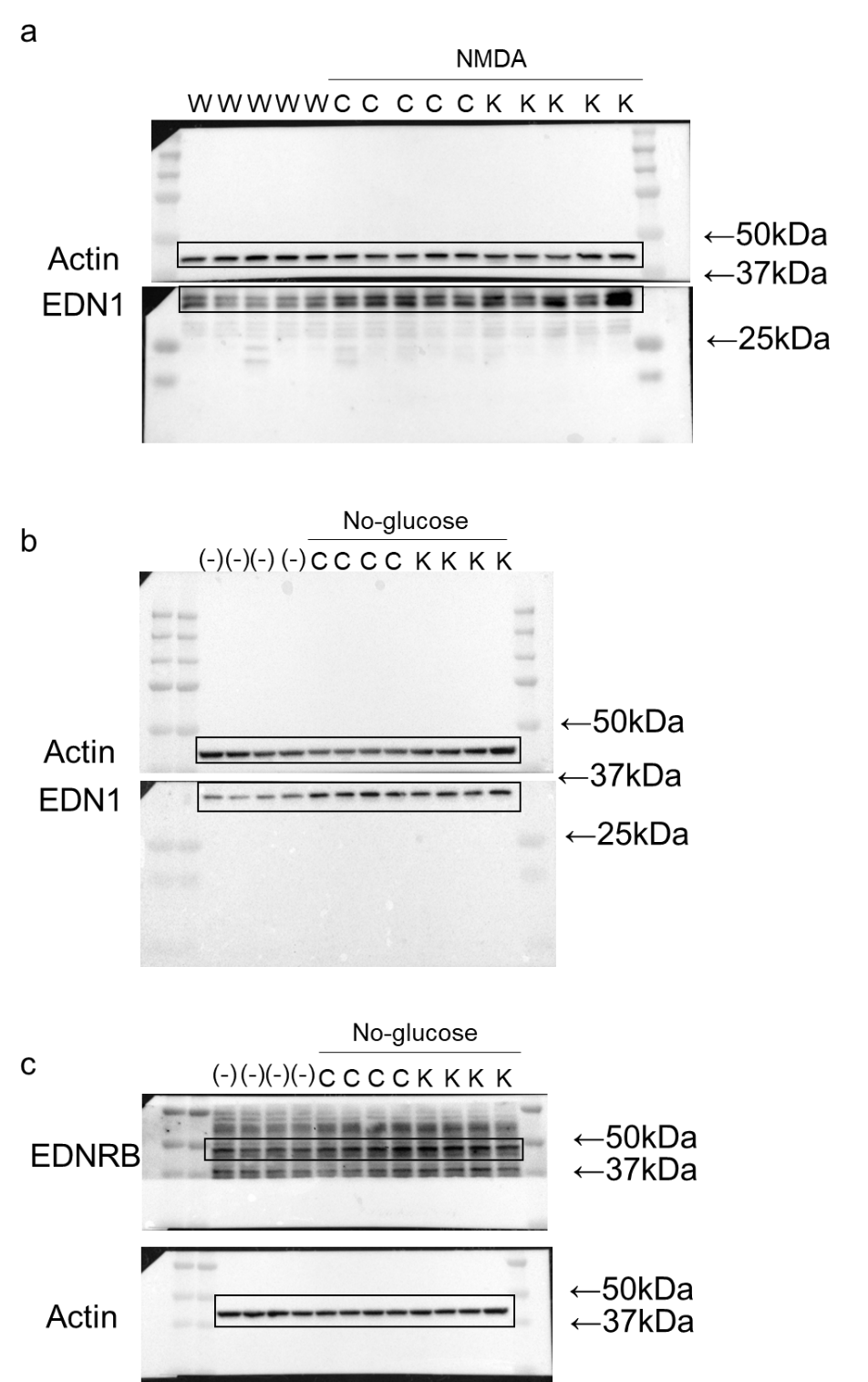
**

**Supplementary Figure S2.** **Complete scans of the western blots presented in the Fig. 3a, 4e, and 4f**. (**a**) Complete scans of western blot using an anti-endothelin-1 (EDN1) antibody (shown in Fig. 3**a**). Actin was used as a loading control. The blot was cut at the 37 kDa position, and the upper part was reacted with an anti-actin antibody and the lower part with an anti-EDN1 antibody. W: retina of wild-type non-treated mice, C: NMDA-injected mice, K: NMDA-injected KUS121-treated mice. (**b**) Complete scans of western blot using an endothelin-1 (EDN1) antibody (shown in Fig. 4**e**). Actin was used as a loading control. The blot was cut at the 37 kDa position, and the upper part was reacted with an actin antibody and the lower part with an EDN1 antibody, respectively. (-): 661W cells cultured with high glucose media, C: cultured with glucose-free media without KUS121 for 24 h, K: cultured with glucose-free media with KUS121 (100 µM) for 24 h, (**c**) Complete scans of western blot using an endothelin receptor type B protein (EDNRB) antibody (shown in Fig. 4**f**). Actin was used as a loading control. The blot was cut at 75 and 25 kDa before the antibody reaction to reduce the required amount of antibodies. Since the background signal of EDNRB was too high to delineate the target band well, the membrane smaller than 37 kDa was hidden with naïve paper and exposed. The blot was then stripped and reacted with an anti-actin antibody. (-): 661W cells cultured with high-glucose media, C: cultured with glucose-free media without KUS121 for 24 h, K: cultured with glucose-free media with KUS121 (100 µM) for 24 h.


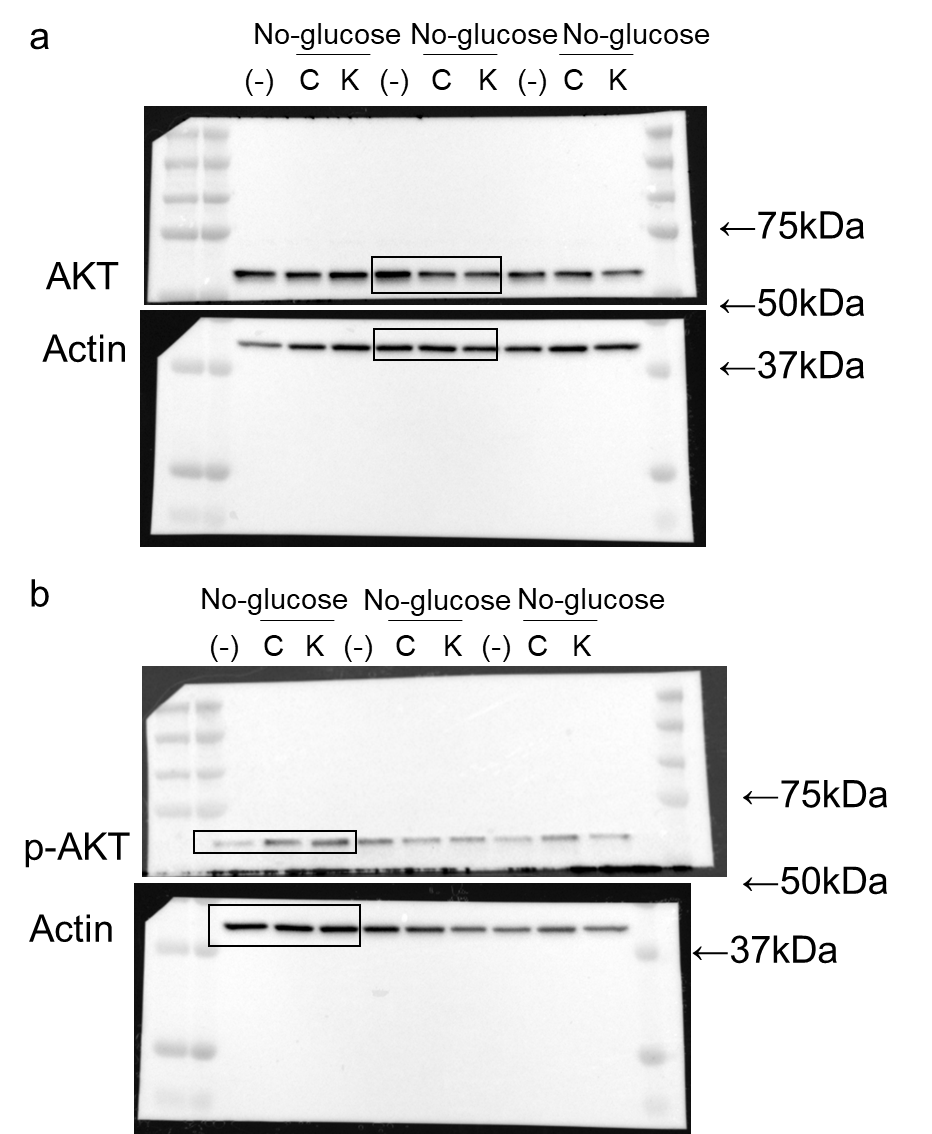


**Supplementary Figure S3. Complete scans of western blots presented in Fig. 8a and 8b.** Protein expression of AKT (**a**) and p-AKT (**b**) in 661W cells was analyzed by western blotting. Actin was used as a loading control. (-): cultured with high-glucose media, C: cultured with glucose-free media without KUS121 for 24 h, K: cultured with glucose-free media with KUS121 (100 µM) for 24 h.

**
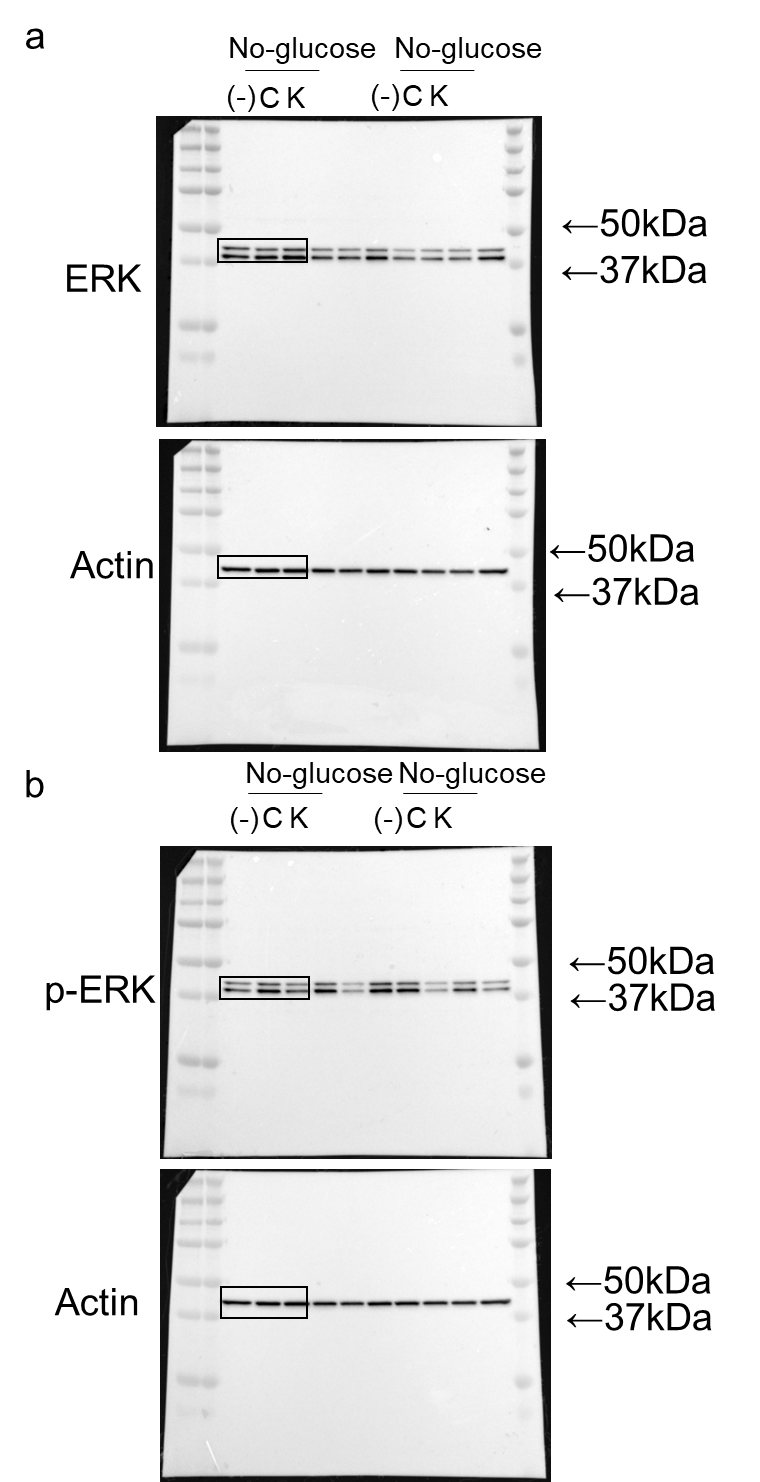
**

**Supplementary Figure S4. Complete scans of western blots presented in Fig. 8e and 8f.** Protein expression of ERK (**a**) and p-ERK (**b**) in 661W cells was analyzed by western blotting. Actin was used as a loading control. (-): cultured with high-glucose media, C: cultured with glucose-free media without KUS121 for 24 h, K: cultured with glucose-free media with KUS121 (100 µM) for 24 h.


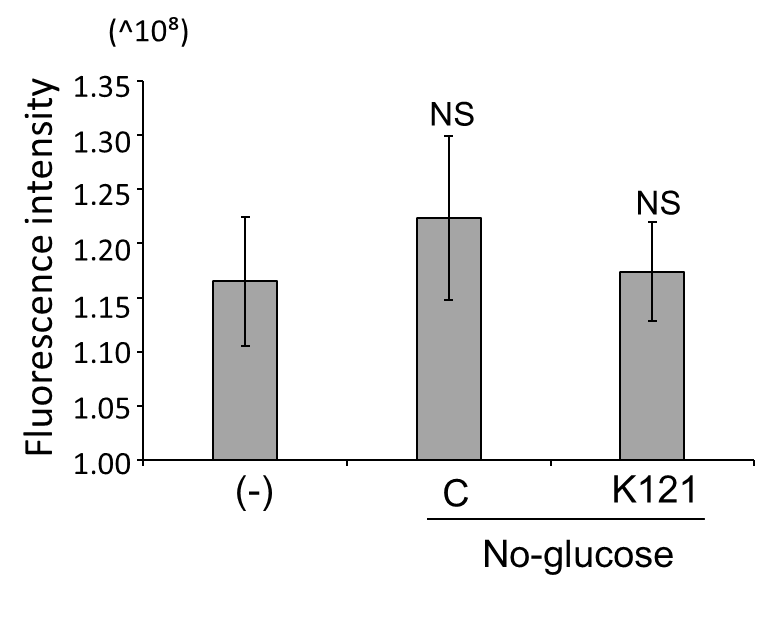


**Supplementary Figure S5. Ednrb function analysis under glucose-free conditions.** CHO cells expressing Ednrb on the cell surface were cultured under glucose-free conditions with or without KUS121 (50 µM) for 16 h, and Fluo8-NW was added. The fluorescence intensity was measured 5 min later with a Nivo Microplate reader. (-): cultured with high-glucose media, C: cultured with glucose-free media without KUS121, K121: cultured with glucose-free media with KUS121. NS: no significant difference compared to (-), Tukey’s HSD.
